# Clinical and molecular response to vorasidenib in post-transplant patient with IDH2-mutant intrahepatic cholangiocarcinoma

**DOI:** 10.1093/oncolo/oyag228

**Published:** 2026-06-12

**Authors:** Vivian V Altiery De Jesus, Timothy McMahon, Sara E Young, Daniel J Zabransky, Mark Yarchoan, Marina Baretti

**Affiliations:** Sidney Kimmel Comprehensive Cancer Center, Johns Hopkins University School of Medicine, Baltimore, MD, 21231, United States; Department of Radiology, Johns Hopkins University, Baltimore, MD, 21231, United States; Department of Cellular & Molecular Medicine, Johns Hopkins School of Medicine, Baltimore, MD, 21231, United States; Sidney Kimmel Comprehensive Cancer Center, Johns Hopkins University School of Medicine, Baltimore, MD, 21231, United States; Sidney Kimmel Comprehensive Cancer Center, Johns Hopkins University School of Medicine, Baltimore, MD, 21231, United States; Sidney Kimmel Comprehensive Cancer Center, Johns Hopkins University School of Medicine, Baltimore, MD, 21231, United States

**Keywords:** Cholangiocarcinoma, IDH2, Vorasidenib, ctDNA, Liver Transplantation, Precision Oncology

## Abstract

**Background:**

IDH2 mutations occur in a small subset of intrahepatic cholangiocarcinoma and currently lack approved targeted therapies.

**Case Presentation:**

We report a post-transplant patient with IDH2-mutant intrahepatic cholangiocarcinoma who developed recurrent disease following multiple treatments for hepatocellular carcinoma and subsequent liver transplantation. Comprehensive genomic profiling revealed an IDH2 p. R172K mutation. Given limited treatment options and contraindications to immunotherapy, off-label treatment with the dual IDH1/2 inhibitor vorasidenib was initiated.

**Results:**

The patient achieved a durable radiographic and molecular response, with a reduction in circulating tumor DNA and a partial response by RECIST criteria sustained for approximately 1 year.

**Conclusion:**

This case highlights the potential clinical relevance of IDH-directed therapy in IDH2-mutant cholangiocarcinoma and demonstrates feasibility in an immunosuppressed post-transplant setting. These findings are hypothesis-generating and support further evaluation of IDH inhibition in this population.

Key Points

- IDH2-mutant cholangiocarcinoma lacks approved targeted therapies.
- Off-label vorasidenib demonstrated durable radiographic and molecular (ctDNA) response lasting approximately 1 year.
- Concurrent everolimus introduces potential biologic plausibility for synergy but limits attribution of response to IDH inhibition alone.
- This case demonstrates durable clinical and molecular response despite prior transplant and ongoing immunosuppression.

## Patient story

A 57-year-old woman with no significant past medical history presented with abdominal pain and a palpable right upper quadrant mass. MRI showed a 9 × 11.3 × 12 cm liver lesion in segment 4 with non-enhancement and invasion of the left portal vein and abutment of the gallbladder. Tumor markers were not elevated (AFP 7.1 ng/mL, CA19-9 15 U/mL, and CEA 0.7 ng/mL). Liver biopsy revealed well-differentiated HCC. She underwent transarterial chemoembolization (TACE) 3 times. She then started on sorafenib, a multikinase inhibitor, but discontinued after 2 doses due to urticaria. Subsequently, she received transarterial radioembolization with radioactive Y90 (TARE Y90) twice followed by single-agent anti-PD1, nivolumab, 3 mg/kg IV every 2 weeks, completing 15 cycles. After the last dose of nivolumab, her tumor was deemed resectable and underwent left partial hepatectomy and cholecystectomy. Pathology revealed a solitary, moderately differentiated (grade 2) 10.5 cm mixed histology of HCC/cholangiocarcinoma (CCA), without evidence of invasion or metastasis, but with positive margins. She received adjuvant 5040 cGy radiotherapy to the hepatic resection margin and adjuvant capecitabine for 3 months.

A year later, a routine MRI abdomen revealed three new hepatic lesions in segment 7 confirmed as recurrent well-differentiated HCC. At the time of her evaluation, the only tumor marker elevated was CEA (4.3). Multidisciplinary evaluation deemed her a liver transplant candidate. She underwent a fourth round of TACE and IR microablation twice. She also received a short course of Nivolumab but it was complicated by autoimmune pancreatitis, requiring hospitalization. Ultimately, she received a hepatitis C-positive liver transplant.

**Figure 1:**
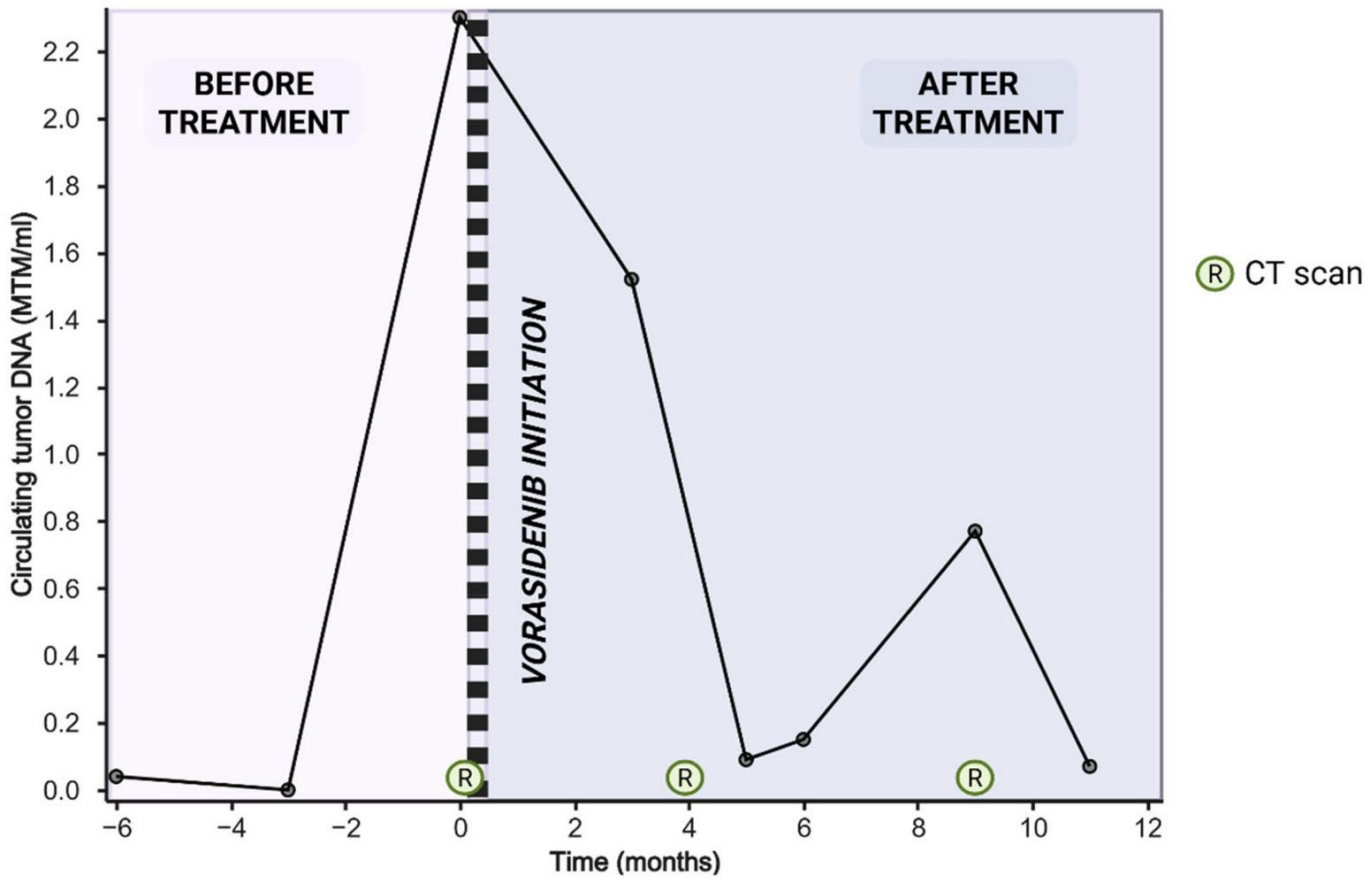
Circulating tumor DNA (ctDNA) levels in peripheral blood (MTM/mL) before and during vorasidenib treatment. Month 0 represents the time of biochemical and radiographic progression immediately preceding vorasidenib initiation. ctDNA surveillance intervals varied based on clinical assessment rather than fixed every-3-month sampling. Circled “R” symbols indicate radiographic assessments by CT imaging. Undetectable ctDNA was defined as 0.00 MTM/mL.

Five years after transplant, MRI showed increased conspicuity of an enhancing nodular lesion in the right posterior hepatic lobe in segment 7. Liver biopsy revealed morphological HCC but immunoprofile supporting CCA differentiation. A subsequent biopsy confirmed pure intrahepatic CCA. Comprehensive genomic profiling of the tumor (Tempus) revealed an IDH2 p. R172K missense variant with a 16.9% variant allele frequency (VAF), tumor mutation burden (TMB) of 11.1 mut/Mb, and microsatellite stable (MSS) disease. No other significant mutation was identified. A repeat biopsy showed moderately differentiated CCA. Concurrently, her Natera ctDNA increased from undetectable to 2.30 MTM/mL (Figure 1).

The initial decision was to proceed with surgical resection. However, intraoperatively, multiple lesions were found in the left lobe of the liver, and resection was aborted. Furthermore, a CT scan was concerned for increasing mediastinal lymphadenopathy. In the setting of her comorbidities, treatment, and transplant, she was not a candidate for a checkpoint inhibitor-based approach, and given the molecular profile, vorasidenib 40 mg daily off-label was started.

The patient provided consent to publish this case report (IRB 00310629).

## Molecular tumor board

Genomic findings, prior therapies, and clinical context were integrated to guide treatment selection in this case. The identification of an IDH2 p. R172K mutation in the absence of approved targeted therapies prompted consideration of off-label IDH-directed therapy. Given the patient’s transplant status and immunosuppression, checkpoint inhibitor-based approaches were not feasible. Based on the molecular profile and available clinical data, initiation of the dual IDH1/2 inhibitor vorasidenib was pursued.

## Genotyping results and interpretation

Comprehensive genomic profiling revealed an IDH2 p. R172K mutation with a VAF of 16.9%, MSS disease, and TMB of 11.1 mutations/Mb. IDH2 mutations promote oncogenesis through the production of 2-hydroxyglutarate and downstream epigenetic dysregulation.

## Functional and clinical significance

Mutations in isocitrate dehydrogenase (IDH) genes represent recurrent oncogenic drivers across multiple malignancies. IDH 1 and 2 (IDH1/2) are NADP^+^-dependent enzymes involved in cellular metabolism and redox homeostasis.^1^^,^^2^ Gain of function mutations in either IDH1 or IDH2 in the cancer cell lead to aberrant production of an oncometabolite, 2-hydroxyglutarate (2-HG).^2^ Although the mechanisms by which 2-HG promotes tumorigenesis remain incompletely defined, known downstream effects include epigenetic dysregulation through inhibition of α-ketoglutarate-dependent enzymes, such as histone demethylases and ten-eleven translocation (TET) family dioxygenases, as well as alterations in the tumor immune microenvironment.^2^^,^^3^

In CCA, the IDH1-specific inhibitor ivosidenib is approved for patients with IDH1^mut^ CCA on the basis of improvements in progression-free survival (PFS). However, oncogenic IDH2 mutations occur in approximately 2%-5% of intrahepatic CCAs, most commonly involving codon R172 substitutions such as R172K and R172S.^4–6^ Because the molecular activity of ivosidenib is restricted to tumors with IDH1 mutations, patients with IDH2-mutant CCA currently lack an approved IDH-directed targeted therapy treatment option.

Two FDA-approved agents with IDH2 inhibitory activity are available for other malignancies: vorasidenib, a dual IDH1/2 inhibitor approved for patients with grade 2 IDH-mutant astrocytoma or oligodendroglioma following surgical resection, and enasidenib, a selective IDH2 inhibitor approved for adults with relapsed or refractory IDH2-mutated acute myeloid leukemia.^7^^,^^8^ Whether IDH2 targeted therapy is efficacious in patients with CCA is not known. Although IDH1 and IDH2 share core oncogenic mechanisms including the production of 2-HG, there are intrinsic differences in IDH1 and IDH2 cellular locations in the tumor cell, and resultant differences in mitochondrial oxidative phosphorylation and cytosolic NADPH pathways.^9^ Here, we report the clinical and molecular response of intrahepatic cholangiocarcinoma harboring an IDH2 mutation treated with the dual IDH1/2 inhibitor vorasidenib.

## Therapeutic strategy

CCA is a lethal malignancy that can manifest at different sites within the biliary tree. It originates from the bile duct epithelial cells and is classified based on its anatomic location. Its incidence and mortality rates have been increasing worldwide in recent decades, coupled with limited treatment options.^10^^,^^11^ Combined hepatocellular-cholangiocarcinoma (cHCC-CCA) accounts for approximately 10% of primary liver cancers and is characterized by substantial histologic and molecular heterogeneity.^12^^,^^13^ Prior reports have demonstrated variable histology at recurrence, including pure HCC, cHCC-CCA, and pure CCA phenotypes.^14–16^ Experimental models further support this plasticity, with hepatocytes demonstrating the capacity to acquire cholangiocytic features under specific oncogenic pressures.^17^ In this patient, the tumor evolved from an initially well-differentiated HCC to cHCC-CCA and ultimately to moderately differentiated intrahepatic CCA harboring an IDH2 p. R172K mutation. Given that IDH2 mutations are rare in conventional HCC,^18^ these findings may reflect underlying lineage plasticity, clonal evolution, or sampling heterogeneity within a combined hepatobiliary malignancy.

This case report adds to the emerging clinical evidence supporting IDH-directed therapy in IDH2-mutant CCA, particularly in patients lacking approved targeted options. It is important to note that IDH2 mutation in CCA is among the top 20 most mutated genes and is also thought to play a role in IDH1 inhibition resistance.^19^^,^^20^

A systematic literature review was conducted to identify published clinical reports of IDH2-mutant CCA treated with IDH2-targeted therapy, including selective IDH2 inhibitors and dual IDH1/2 inhibitors (Table 1). Articles were excluded if they only had IDH1-only cohort without IDH2 subgroup data, preclinical studies, or a review without primary patient data. Across published reports, a total of 34 patients with IDH2-mutant CCA have been treated with IDH-directed therapy; however, clinical experience remains limited and RECIST-assessable outcomes are inconsistently reported^21–23^ (Table 2). Therefore, a pooled ORR was not calculated. Available data suggest modest activity of IDH-directed therapy in this population, with disease control reported in early-phase studies and limited PFS data where available. These findings underscore the need for prospective evaluation of mutant inhibition in IDH2-mutant CCA.

**Table 1:** Reported clinical experience with IDH2-directed therapy in IDH2-mutant cholangiocarcinoma.

Study design treatment setting	CCA population (n)	IDH2-mutant CCA receiving IDH Inhibitor (n)	Treatment	Response rate in IDH2 mutant CCA	PFS in IDH2 CCA	Notes
**^a^Phase I^24^ Advanced solid tumor**	CCA mutation subtype not stratified (24)	Unknown	**Vorasidenib** monotherapy (dual IDH1/2 inhibitor)	Not reported		mPFS 1.9 months reported for overall non-glioma cohort
**Phase I^21^ Advanced/unresectable**	IDH1- or IDH2-mutant CCA Monotherapy cohort (58)	25	**LY3410738** monotherapy (dual IDH1/2 inhibitor)	ORR 4% (1 PR)	mPFS 3.1 months (95% CI: 1.9-3.7)	60% DCR reported separately
**Single-center retrospective case series^23^ Advanced/unresectable**	IDH2-mutant intrahepatic CCA (6)	1	**Enasidenib** + chemo/IO (selective IDH2 inhibitor)	Not reported		Time on therapy 18.1 months
**Multi-institutional retrospective^22^ Later-line (advanced disease)**	IDH2-mutant CCA (33)	8	**Enasidenib** monotherapy (selective IDH2 inhibitor)	Not reported	mPFS 3.9 months (CI 1.1-5.1)	
**NCT02273739**	IDH2-CCA (8)	Not reported	**Enasidenib monotherapy (selective IDH2)**	Not reported	Not reported	Tumor-specific RECIST outcomes not publicly reported

a The study included patients with cholangiocarcinoma; however, the number receiving vorasidenib and their specific responses are unknown because results were reported within the non-glioma cohort.

**Abbreviations:** CCA, cholangiocarcinoma, chemo/IO, chemoimmunotherapy; CI, confidence interval; CR, complete response; DCR, disease control rate; mPFS, median progression-free survival; ORR, overall response rate; PD, progressive disease; PR, partial response; SD, stable disease.

**Table 2:** Radiographic response assessment according to RECIST 1.1.

Time from treatment initiation	Sum of target lesion diameters (mm)	Change from baseline (%)	RECIST 1.1 category
**Baseline^a^**	56	—	—
**2 months**	56	0	Stable disease
**7 months**	42	−25	Stable disease
**9 months**	39	−30	Partial response
**11 months**	38	−32	Partial response

a Baseline before vorasidenib initiation.

The first study is a phase I clinical trial testing vorasidenib in patients with IDH1/2-mutant solid tumors, including 24 CCA in the non-glioma cohort. There is no specific response reported for cholangiocarcinma subgroup.^24^ The second study is a phase I study of LY3410738 an oral, brain-penetrant, dual IDH1/IDH2 inhibitor in solid tumor.^21^ A total of 58 patients with IDH1/2 mutant relapsed/refractory CCA were treated as monotherapy or combination with gemcitabine, cisplatin, and durvalumab.^21^ The IDH2 mutant (*n* = 25) were reported as monotherapy with an overall response rate of 4% and stable disease for 56% in patients with CCA harboring IDH2 mutations (*n* = 25).^21^ A single-center retrospective study looked at 6 patients with IDH2 mutant CCA, only one received enasidenib for 18.1 months in combination with their immunochemotherapy; RECIST response was not reported.^23^ Lastly, a multi-institutional retrospective study of IDH2-mutated CCA found that 8 out of 33 patients received enasidenib as Nth line therapy with a median PFS 3.9 months.^22^ Collectively, these findings underscore the need for broader clinical evaluation of mutant IDH inhibition in CCA harboring a mutation in IDH2.

Vorasidenib was selected based on several clinical and biologic considerations. As a dual IDH1/2 inhibitor, vorasidenib provides broader isoform coverage compared with selective IDH2 inhibition and has been evaluated in solid tumor populations, including CCA. In contrast, enasidenib has primarily been studied in hematologic malignancies, with limited published efficacy data in biliary tract cancers. Additionally, in the setting of prior transplant, evolving histology, and limited standard therapeutic options, vorasidenib represented a rational targeted approach with a manageable safety profile relative to traditional cytotoxic chemotherapy. Following initiation of therapy, the patient demonstrated both radiographic improvement and a decline in ctDNA levels.

Notably our patient was post-transplant on everolimus, an mTOR inhibitor. IDH inhibition escape is believed to be through PI3K/AKT/mTOR signaling.^25^ There are no published clinical studies showing synergy between mTOR inhibitor and IDH2 inhibitor in CCA, but it is biologically reasonable. The contribution of concurrent everolimus to the observed response cannot be excluded, and potential biologic synergy between mTOR inhibition and IDH-targeted therapy remains hypothesis-generating. Vorasidenib was administered with close monitoring for hepatic toxicity and potential drug-drug interactions, particularly given concurrent everolimus use and shared CYP3A4 metabolic considerations; however, no clinically significant interaction or dose-limiting toxicity was observed during treatment. Importantly, this case highlights the feasibility and tolerability of IDH inhibition in an immunosuppressed post-transplant patient, a population excluded from prospective clinical trials. Moreover, hepatic safety considerations for IDH inhibition in CCA, particularly in immunosuppressed transplant recipients, remain undefined, as existing monitoring recommendations derive primarily from glioma and acute myeloid leukemia (AML) populations.

To our knowledge, this represents one of the first reported confirmed RECIST partial responses to vorasidenib in IDH2-mutant CCA in the posttransplant setting. To this day, our patient continues to sustain durable disease stability for over a year.

## Patient update

**Figure 2:**
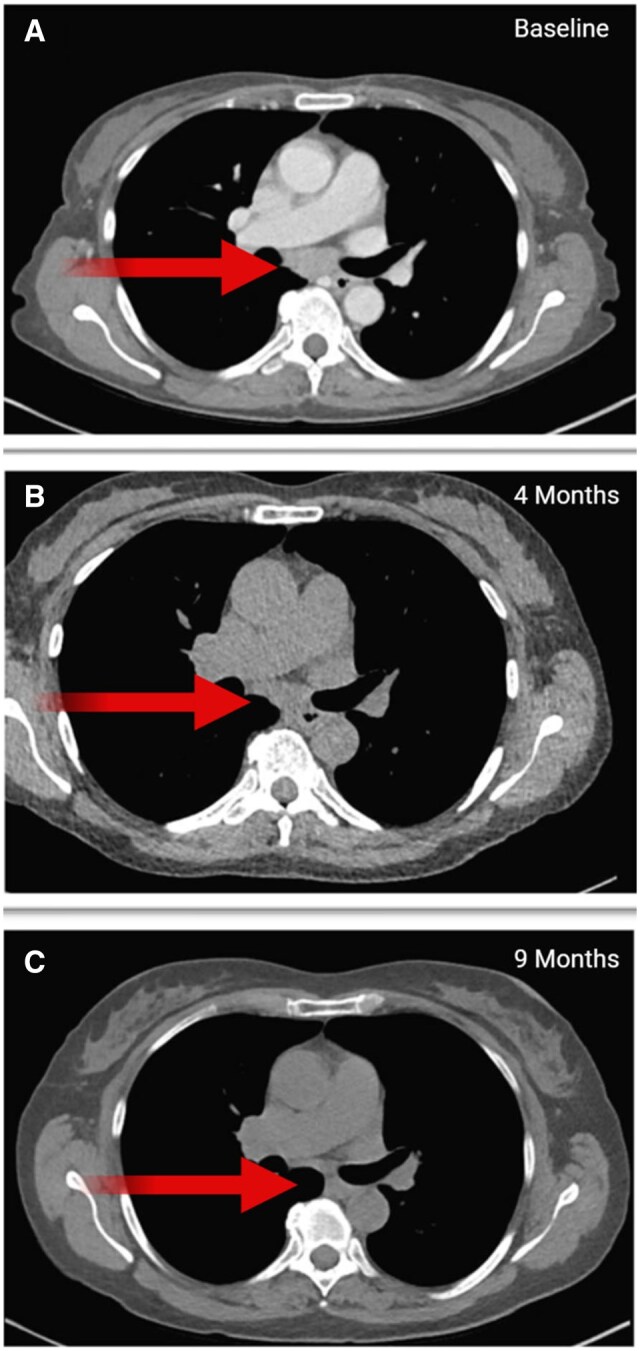
Vorasidenib radiological response in the subcarinal lymph node. Radiographic response to vorasidenib therapy. (A) Baseline CT chest before the initiation of vorasidenib demonstrating progressive mediastinal lymphadenopathy. (B) CT chest obtained 4 months after the initiation of vorasidenib demonstrating interval decrease in mediastinal lymphadenopathy. (C) CT chest obtained 9 months after the initiation of vorasidenib demonstrating sustained stability of the subcarinal lymph node and overall mediastinal disease burden.

A month after initiation of vorasidenib, ctDNA decreased to 1.52 MTM/mL and has continued to decrease since then (Figure 1). Target lesions were selected and measured according to RECIST 1.1 criteria, with consistent longitudinal assessment of the sum of diameters. Radiologic assessment demonstrated early disease control at 2 months, with stable disease by RECIST 1.1 (0% change from baseline in the sum of target lesion diameters). A repeat liver biopsy performed 5 months after initiation of vorasidenib to reassess histology and molecular features in the setting of evolving disease behavior confirmed persistent well-differentiated CCA. Tempus assay showed known IDH2 pR172K missense variant with a 22.9% VAF, TMB 10.5 m/MB and MSS. Additional co-occurring genomic alterations included BAP1, FHIT, FOXP1, and PBRM1. At 7 months, further tumor regression was observed, with a 25% reduction in the sum of target lesion diameters from baseline, remaining consistent with stable disease by RECIST 1.1. Partial response was first achieved at 9 months, with a 30% reduction from baseline. Ongoing partial response was confirmed at 11 months, with a 32% reduction from baseline. Clinically, hepatic lesions remained stable, and there was a marked decrease in mediastinal lymphadenopathy (Table 2; Figure 2). The patient’s response is ongoing at the time of this report, approximately a year after initiating therapy.

The patient achieved a durable radiographic response, with partial response by RECIST criteria sustained for approximately one year, accompanied by a marked decline in circulating tumor DNA. Treatment remains ongoing at the time of this report, with continued clinical stability.

## Data Availability

The data underlying this article cannot be shared publicly due to the privacy of individuals who participated in the study. The data will be shared on a reasonable request to the corresponding author.
